# How gen Z can improve community literacy about the 17 SDGs? A realistic approach to construct a futuristic change-maker paradigm

**DOI:** 10.1007/s44173-022-00002-2

**Published:** 2022-05-14

**Authors:** Sima A. Hamadeh

**Affiliations:** 1 Executive & Graduate Studies Department, Northwestern University, Doha, Qatar; 2 Nutrition & Dietetics Sciences Department, Haigazian University, Beirut, Lebanon

**Keywords:** Generation Z, Sustainability literacy, Communication, Science journalism, Society, Future development, Change paradigm, Smart technologies, Environmental management

## Abstract

Many fields and different approaches have undergone a crucial merging of implications and have been co-involved in the developing and/or implementation of the 17-SDGs concepts. However, little has been done on the role of the generation Z who is transforming the workplaces and societies by creating and interpreting trends.

The objectives of this research are to provide perspectives from the world about the generation Z participatory role and supporting contribution among their communities to reaching SDGs, and to illustrate their active role in a Futuristic Change-Maker Paradigm.

A general review was conducted to explore the existing data on governance mechanisms for SDGs implementation regarding the multi-dimensional layers (social, economic, environmental, health, policies, communication, and technology) of the sustainable development structure that need to be considered in a systemic-sensitive approach.

Various channels of youth participation can be associated with different levels of social and communities’ commitment. In this sense, this article; 1) confirms that generation Z could play an active role in utilizing state-of-the-art opportunities to address the established current UN-SDGs agenda, and 2) suggests how communication and science journalism can be conceptualized as a development intervention to go beyond the 2030 SDGs agenda. This study recommends several innovative areas for the integration of generation Z insights and activities as a basis for exploring the potential of this generation to improve communities’ literacy and behaviors about the 17-SDGs.

This study ends with a practical and theoretical consideration to build a Futuristic Change-Maker Paradigm that can be extrapolated to other countries in their advance towards sustainability and SMART environmental management.

## Introduction

*“Have we entered a new phase of planetary history?” and “Is there a prospect for our planet’s future?”*

To answer these questions, it is crucial to define and understand the “Anthropocene” notion and whether we are living a turning point in the geological history transformed by the strained human activity. *Anthropocene* is mainly defined by the concept that the earth has moved into a novel geological epoch characterized by human domination of the planetary system at an unprecedented rate and scale [1]. The rapid population growth in the world, urbanization, pollution, irresponsible agriculture, deforestation, and gigantic technological advancements are some examples of Anthropocene, which have caused peculiar and significant changes to our planet [1, 2].

For the last decades, our planet faces multiple and complex challenges such as loss of biodiversity, climate change, plastic pollution, etc. [1, 2]. Our awareness of the current state of the planet and the effects of our activities is a key factor to take innovative and comprehensive actions to build a better sustainable future for the generations and to save the earth [2, 3]. Therefore, developed and developing countries need to adopt and implement the United Nations new agenda including the 17 Sustainable Development Goals (SDGs) entitled “Transforming our World: The 2030 Agenda for Sustainable Development”. An agenda that calls for action by targeting 5 areas of critical importance, the so-called 5Ps: people, planet, partnership, prosperity, and peace [4–6]. Besides, it recognizes the significance of adapting a people-centered, universal, transformative, and integrated approach to have a better healthy planet for future generations [2, 4, 6, 7].

Many fields and different approaches have undergone a crucial merging of implications and have been co-involved in the developing and/or implementation of the 17-SDGs concepts [6]. The issue needs to be addressed from multiple angles, including providing sustainable, ecological, socio-economic, and nutrition literacy to individuals, but equally important building the capacity of supply chains specifically a green food chain to deliver more responsible products and accountable food [3]. Patently, the COVID-19 pandemic had a huge and global effect on peoples’ lifestyles and their environments strongly impacting the nutrition and food security related-matters [8]. For instance, in a matter of weeks, the accessibility, availability, and sometimes the affordability of basic foods and daily practices changed radically. The anticipating recovery from the pandemic should be the opportunity to build back better for the future and to transform our lives, cities, and food systems to be more sustainable and in the direction of achieving the SDGs 2030 agenda [3]. Therefore, adopting digital solutions and using online platforms for e-commerce or delivery services during the pandemic have forced people to reconceptualize the way they run their production and businesses, and thus help to improve food supply chains by offering longer-term benefits [8, 9].

Lately, literature review of scientific and grey data identified the particular and significant role of youth in achieving this historic new agenda by 2030 [10–13]. More specifically, the impact of the active participation of youth in their communities that must go hand-in-hand with a plan that builds socioeconomic growth and addresses a range of health and social needs, while tackling sustainability literacy, climate change, and other agricultural and environmental issues [12–14]. However, little has been done on the role of the generation Z who is transforming the workplaces and societies by creating and interpreting trends [15–18].

Although our planet has never been in such a hazardous state as it is today, but we have also never been better equipped with human competences and necessary tools to understand what is happening and what needs to be done among distinct segments of the population and in different contexts. The objectives of this research are first, to provide perspectives from the world about the generation Z participatory role and supporting contribution among their communities to reaching SDGs, and second to illustrate their active role in a Futuristic Change-Maker Paradigm.

## Methodology

To answer the research objectives, a general review was conducted to explore the existing data on governance mechanisms for SDGs implementation regarding the multi-dimensional layers (social, economic, environmental, health, policies, communication, and technology) of the sustainable development structure.

With the help of traditional desk research such as literature and web searches, and data mining, the latest SDGs’ matters coverage in scientific peer-reviewed articles, grey data, books, countries’ reports, international reports (United Nations Framework Convention in Climate Change-UNFCC, United Nations Environment Program-UNEP, Food and Agriculture Organization-FAO, World Meteorologic Organization-WMO, World Health Organization-WHO, etc.), magazines, and newspapers was examined in this review to shed light on the types of manuscripts and/or activities content that can be rightfully used in identifying the role of youth in improving sustainable literacy in their communities.

Finding from several studies showed the importance of considering such multifaceted collected data in a systemic-sensitive approach [3, 19, 20]. Therefore, the systemic approach was used to analyze with a global point of view the complex and multi-dimensional structure of the sustainable development in different systems such as agroecological, biophysical and sociotechnical systems. Implementing such systemic approach helps: 1) to understand the system complexity and the interactions between its components; 2) to identify all emerging factors and properties specific to the research subject matters; and 3) to support the conception and communication of the complexity through models and paradigms [3, 20].

Moreover, the sensitive approach was used for a holistic analysis of how any initiative, program and policy will thrive through data-driven forecasting [3, 19]. For instance, the incorporation of real-time data flows: 1) to make informed decisions related to this research subject matters; and 2) to also create dynamic actions that are predictive in determining the direction of future trends.

## Results

Our findings indicate that the advanced analysis approach used in this study helped to provide better insights after linking the literature review from all types of narratives with the proposed and forecasting *“Futuristic Change-Maker paradigm”.* Results from scientific peer-reviewed journals, national and international reports, public sectors programs, private sector activities, civic sector initiatives, magazines, newspapers, and social media posts were amalgamated, thoroughly analyzed, and presented in the following 4 subsections.

### United Nations developments goals: from MDGs to SDGs

Throughout the history, the concept of “*development*” has submitted a relevant change from economic development to human development, then to sustainable human development (SHD), which is defined by expanding the capabilities and substantive freedoms that people enjoy without compromising those of future generations [21, 22]. Although, the SHD is the core of an extensive development paradigm, but it worked on several concepts separately such as ecology, global citizenship, human development, cooperation, and coordination [23]. Then the challenge was clearly to care about all developments concepts harmonically and to build up structures that support this new comprehensive and integrated paradigm [4]. In this sense, the United Nations developments goals managed to put them together in a relationship first in 2000 with the Millennium Development Goals (MDGs), and second to make them more interdependent in 2015 with the SDGs [24].

*“It is a roadmap to ending global poverty, building a life of dignity for all and leaving no one behind. It is also a clarion call to work in partnership and intensify efforts to share prosperity, empower people’s livelihoods, ensure peace and heal our planet for the benefit of this and future generations,” underscored UN Secretary-General Mr. Ban Ki-moon, September 2015.*

**Fig. 1:**
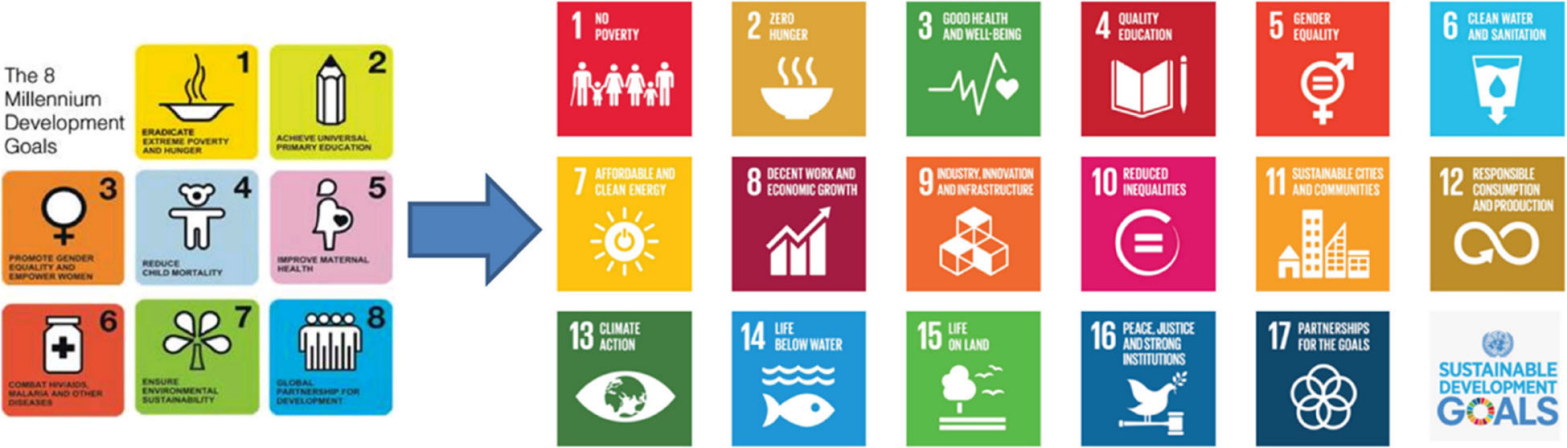
Adapted from the Nakatani H, Report 2016 [5]

The United Nations Development Programme (UNDP) is the leader in this global movement and collaborates with other organizations to help all countries make the development goals a reality and timely [24, 25]. The MDGs developed in 2000 were simple, clear, and limited, where health occupied the central position and their targets were relatively quantitative such as reducing child mortality, improving maternal health, eliminate hunger, etc. [5]. In 2015 the MDGs come to the end of their term, and a post-2015 agenda, comprising 17 SDGs, takes their place (201 5-2030) as “Fig. 1” illustrates them.

Nakatani (2016) in his WHO report looked back 15 years (2000–2015) at the existing trends and positive forces during the MDG era and assessed the main challenges that will affect health in the coming 15 years [5]. Based on this assessment, a new transformative approach was adapted by the WHO and the United Nations to produce new plans and strategies aimed at the post-MDG era [25]. Taking these changes in parameters in mind, leaders from 193 countries of the world planned for new sustainable development goals and developed the 17SDGs with different global agenda and broader objectives to target many areas for action including health, climate change, poverty, gender equality, women’s empowerment, capacity and institutional building, planet protection, among other pledges grounded in an integrated cross-sectoral approach [2, 5, 6]. The following table (Table 1) summarizes the main differences between the MDGs and the SDGs.

**Table 1:** Comparison between the Millennium Development Goals and the Sustainable Development Goals

MDGs 2000–2015	SDGs 2015–2030
More narrow - 8 isolated goals - Great attention to health matters - Limited attention to sustainability	More inclusive - 17 integrated goals - Based on sustainability in all its 3 dimensions (social, economic, environmental)
Focus on the South (relevant to the developing countries or to the Official Development Assistance “ODA” communities)	Universal (relevant to all countries but adaptable to different contexts)
Means of implementation: limited to North-South finance	Means of implementation: include the following - Market access - Technology transfer - Capacity building - Policy support
Weak reporting follow-up & review	Robust global framework for monitoring, follow-up & review

This new transformative approach and integrated agenda used for the development, implementation, and evaluation of the global goals helped the 193 countries that signed the 2030 agenda in their efforts to bring the SDGs to life by: 1) establishing enabling sustainable environments at different levels (governance, institutions, policies) grounded in a sound evidence-based data, 2) raising public awareness, 3) seeking engagement of different and multidisciplinary key stakeholders, 4) addressing interconnected challenges in a comprehensive way bringing together multiple actors, and 6) adapting the SDGs to local and national contexts [2, 5, 7, 23].

### Food systems, nutrition and driving progress on the SDGs

**Fig. 2:**
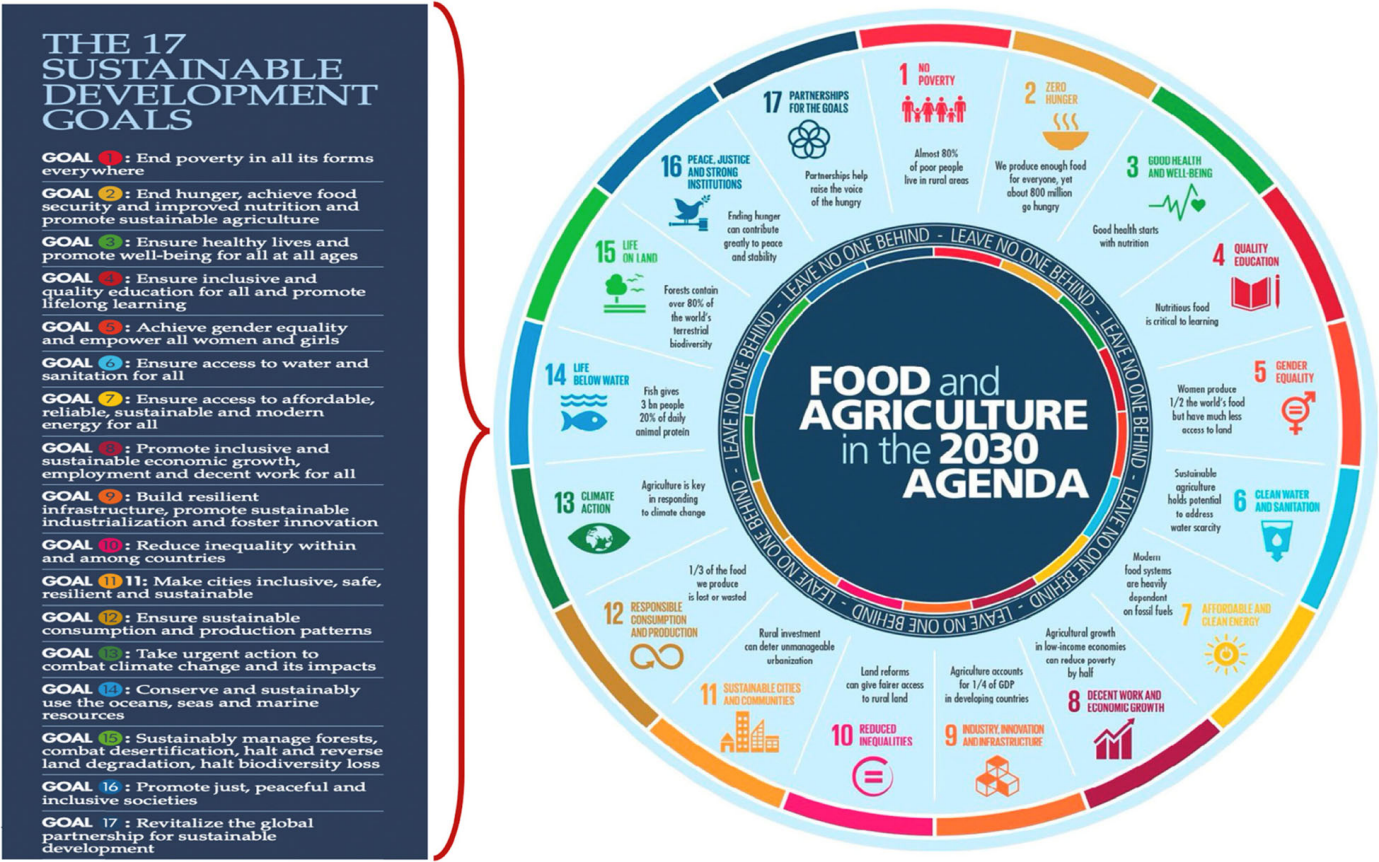
Connecting agri-food systems to the 17SDGs (Wheel of Food & Agriculture in the 2030 agenda adapted from FAO report, 2016) [2]

By assessing the existing data, we found substantial scientific evidence about diets in the *Anthropocene* that links food with people’s health and environmental sustainability [3, 26]. Therefore, a radical transformation of the global food systems towards healthy planetary practices is vital and delaying relevant actions will only intensify the likelihood of serious health problems and environmental consequences [26]. For instance, there are vast co-benefits and opportunities of integrating food supply chain processes and systems (sourcing of raw materials, farming, production, processing and manufacturing, transportation, distribution, and consumption), and nutrition matters across all the SDGs [2, 3, 10]. A map of the connections between food and agriculture as keys to achieving the entire set of the SDGs is summarized in “Fig. 2”.

It is clear that a radical transformation in the global food systems will not occur without a comprehensive operational and evaluative agenda, hard work, political will, sufficient human and financial resources [3, 26]. Besides, multi-level actions guided by solid scientific evidence, and greater cooperation, commitment and communication across sectors are crucial to shift towards healthy and sustainable planet [2, 3, 6, 26].

In order to maximize the opportunity offered by the SDGs global vision of an integrated food system for sustainable development, it is fundamental to transform our ways of thinking and acting to ensure greater public participation and collaboration across the 17 goals [2, 26]. More specifically and trending nowadays, active participation of youth is a necessary precondition to increase community literacy about SDGs and to greatly support their implementation [5, 12, 13]. For instance, the *International Youth Day* observed by the United Nations on August 12 to raise awareness of the need to ensure the creative engagement and active participation of youth to build a better future for all [12]. In this context, the *International Youth Day* in 2021 was addressing a special theme entitled: “Transforming food systems: Youth innovation for human and planetary health” where the United Nations hosted for the first time a *YouthLead Innovation Festival* to highlight revolutionary solutions for the SDGs suggested by young innovators [13]. This festival provided 6 spotlight sessions (Health and wellbeing, Climate action and biodiversity, Food security, Economic empowerment and employment, Digital technology, Education), and a social media platform (TikTok) for youth to share their ideas and solutions [13].

The role of youth, in particular the generation Z, in generating new knowledge and approaches to food systems transformations coupled with SDGs 2030 agenda are presented in the following subsection of the results.

### Generation Z: characteristics, roles, and responsibilities

**Fig. 3:**
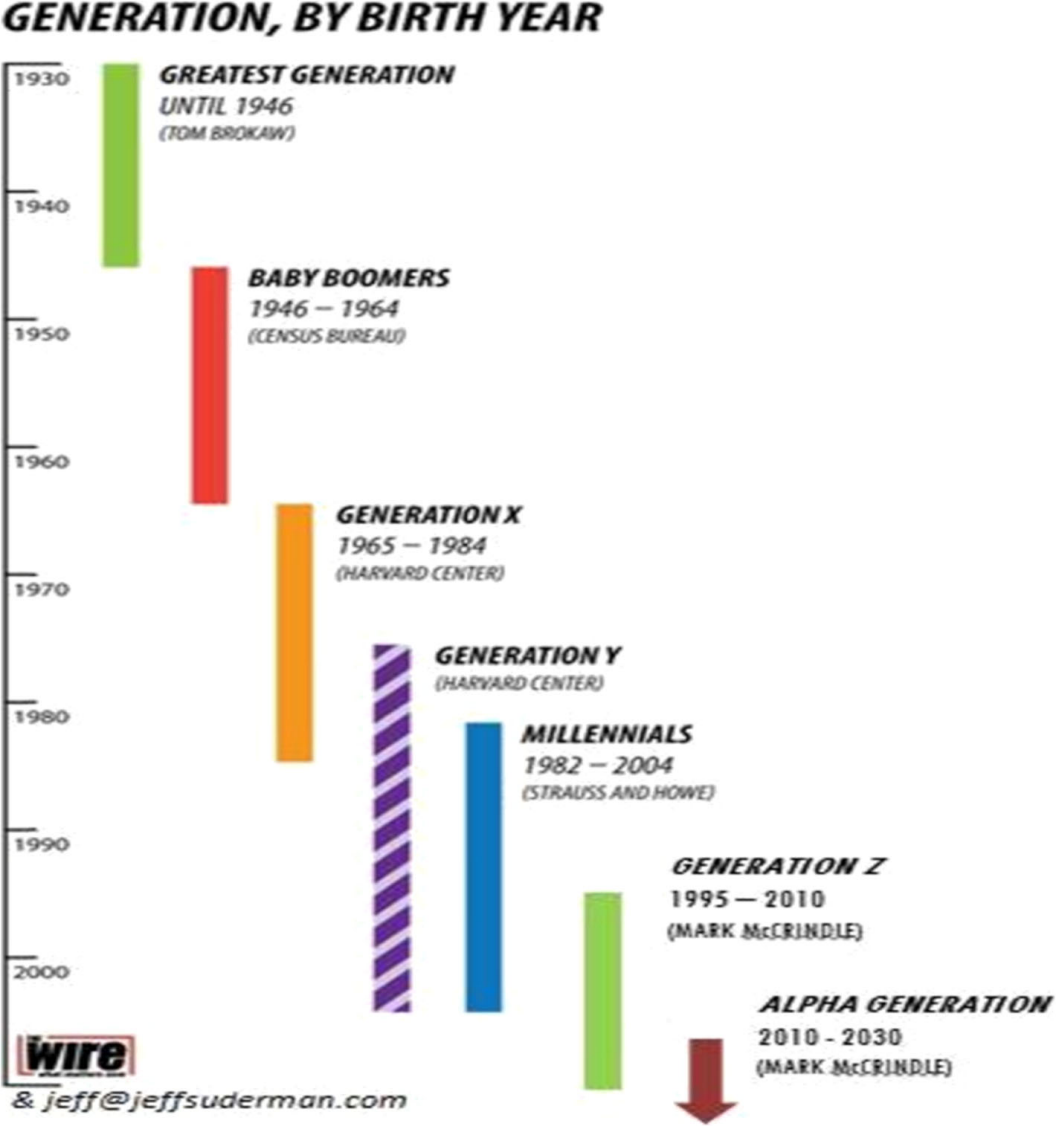
Generations, by birth year. (Adapted from Nagy A and Kolcey A, 2017) [29]

A generation is usually defined by common sociocultural and economic circumstances, and contextual factors [27, 28]. Although, there is a recognizable generational pattern, but this does not mean that every individual belonging to a specific generation will show the same attributes [29]. Moreover, the borders between generations are blurred and the dates used to define different generations are relatively unsystematic even if the Strauss and Howe model refers to the 20 years lifetime for each generation with particular patterns of cyclicality [29–31]. Overall, classifying and naming any new generation as showed in “Fig. 3” is mostly important for global economic and marketing purposes [29, 32].

Among these generations, youth can play a crucial role in the development and improvement of the community literacy of SDGs, and the promotion of the UN agenda 2030 for a better future of our planet. Several findings showed the role and impact of youth on modifying their communities and societies by improving the sustainability literacy, and how the world will change when a new generation leads such as the generation Z [27, 33]. A change that is described a “Youthquake” and a hope for our planet’s future [15, 34].

### Who is generation Z?

Although the emerging literature regarding the generation Z showed some disagreement amid researchers on the exact dates defining it; however, this generation includes those born between 1995 and the early 2010s [14, 30]. Members of this generation are true digital natives and considered, till today, the only group of people raised exclusively with a technology influence and thus, likes immediacy and practicality [33]. They bring same characteristics of the previous generation born in the early 1980s through the mid of 90s “The Millennials” but they also have a lot of differences [28, 33, 35–39]. The most distinctive traits of the digital natives are individualism, addiction to technology, convenience and speed, dependency, and freedom [14, 17, 18].

**Fig. 4:**
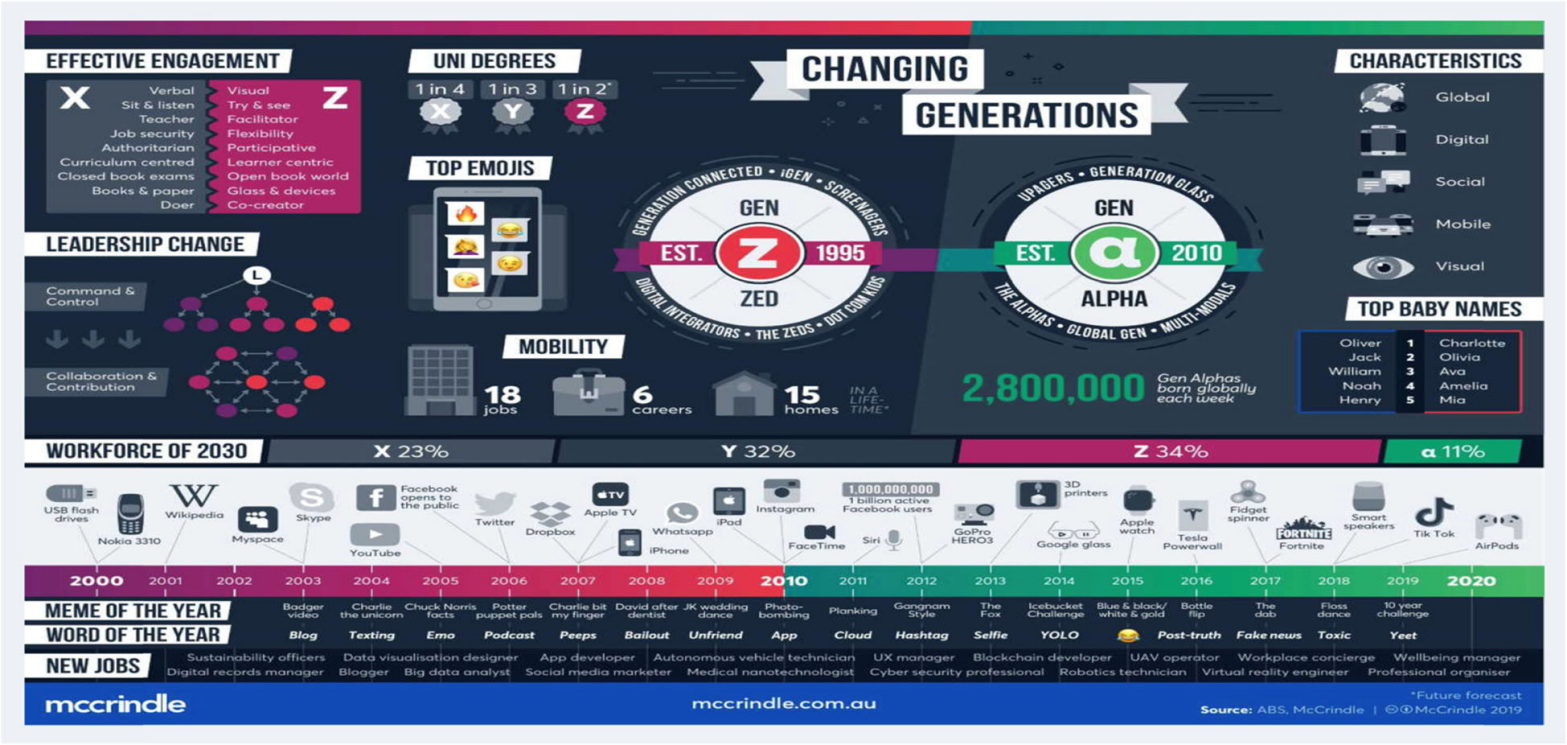
Facts and Stats 1: Gen Z and Gen Alpha infographic update. (Adapted from Mccrindle Report, 2019) [40]

**Fig. 5:**
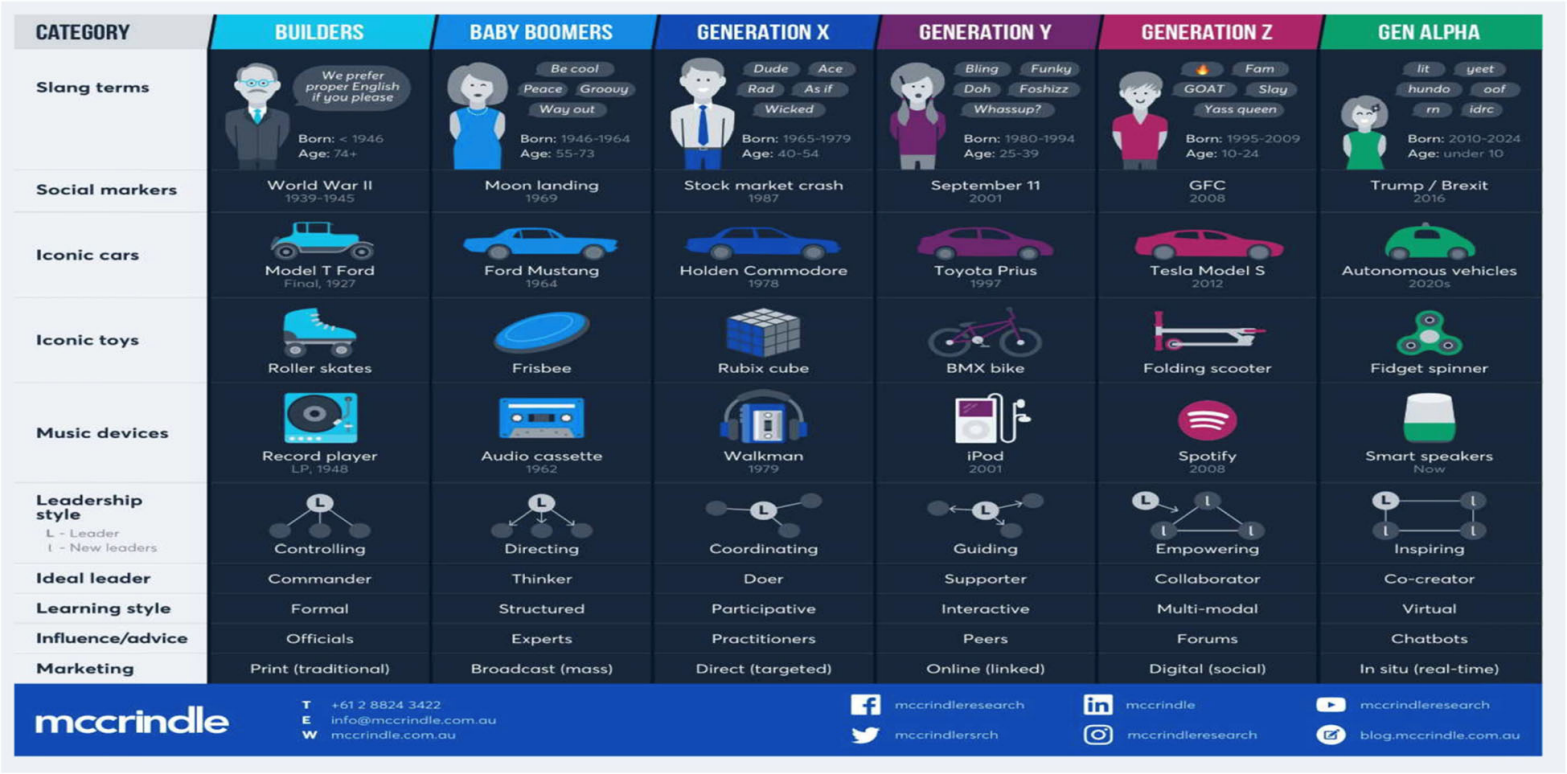
Facts and Stats 2: Gen Z and Gen Alpha infographic update. (Adapted from Mccrindle Report, 2019) [40]

The following adopted infographics from Mccrindle report “Fig. 4 and Fig. 5” showed clearly and in comparison, the general traits of the generation Z such as their education level (the best educated generation yet), leadership styles (empowering style), preferred social media platforms (YouTube, Instagram, TikTok), favored marketing strategies (digital marketing, social media), ideal engagement systems (collaboration), learning styles (multi yet engaging styles), etc. [40].

After examining the literature, several characteristics of Generation Z as students, consumers and workers were identified and summarized in “Table 2” [16, 27, 28, 30, 35–39].

**Table 2:** Summary of generation Z characteristics, needs, expectations, perceptions and aspirations as students, consumers, and workers

Generation Z characteristics		
As students they ask for:	As workers they are:	As consumers they:
Practical & relevant information (engaging, pragmatic, technologically advanced & visual learning)	Resilient & resourceful (more likely to adopt new skills & develop new business models)	Focus on innovation
Utilizing video-based learning	Open-minded & dedicated to inclusivity	Insist on convenience
Incorporating intra-personal learning into class & group work *“Think-Pair-Share”*	Committed to large & global causes	Are result-oriented
Community engagement opportunities to address societal needs	Authentic in their employee experience	Are pragmatical & underly desire for security
Connection to internship opportunities	The most technologically sophisticated generation	Have tendency towards entertainment & distraction

### Why a special attention should be given to generation Z?

Generation Z represents a sizeable group entering the college and workplace and comprising 34% of the population by 2030 [30, 35, 40]. Therefore, identifying the distinctive attributes and the generational influences of this youth group can assist SDGs adopters in successfully engaging them as key actors to promote the 2030 agenda and to spread the sustainability literacy in their communities at all levels [41]. Based on the specific characteristics of the generation Z presented in this review, they are expected to be creative in their workplaces, entrepreneurs, and multitasking individuals [14, 17]. In another example, Hansen and Wyman (2021) showed that generation Z has developed interest in social responsibility and particularly in sustainability matters [42] and therefore, it is increasingly likely that these digital natives would use their entrepreneurship characteristics to seek change in their environments to achieve the SDGs agenda by 2030 [17].

While data and verdicts regarding this generation is still emerging and generational stereotypes are not complete, offering a specific social, educational, and professional support to members of generation Z in their societies, institutions, workplaces, leisure times, and activities is necessary to make a remarkable change in our lives and to our planet [13, 33, 35]. Therefore, the undermentioned section considers reexamination of teaching-learning approaches, working modalities and systems thinking as generation Z rapidly infiltrates colleges and workplaces where they can make a big difference towards the SDGs 2030 agenda.

### A futuristic change-maker paradigm

This research reflected on the evolution of the *sustainability literacy* concept and how it can be re-explained under the umbrella of the SDGs. Besides, the historical evolution of the complex concept of SDGs has been shown and has been justified its approach from the youth community context, primarily from the generation Z perspective. Outcomes from past research and activities done in this field were mostly documents and recommendations to; 1) share information and facts, 2) to report periodically all initiatives and results, 3) to adopt measures and policies on mitigation, 4) to work on and reach the SDGs, and 5) to develop corporate social responsibility-CSR activities [2, 10, 25, 43]. Nevertheless, additional lessons were learned from the literature and how new areas of activities should be explored to achieve the SDGs in every context and society such as arts activities (SDGs themes in: RAP music lyrics, drama pieces, animated artworks, etc.), social media platforms (sharing stories or broadcasting messages about SDGs), global citizens stories (influencers, Youtubers, bloggers, etc.), and in science events, podcasts, high-level panels, and policies corridors where Generation Z should play a part [3, 25, 44, 45]. In this sense, creativity, skills, literacy, and the knowhow are fundamental to achieve the SDGs. Moreover, it demands capacity building, data accountability, alliances with multi-sectoral stakeholders, and availability of resources (human, financial, technology) [3, 25, 46].

In other words, embracing such measurements in the nations’ 2030 agenda where generation Z, can use their potentials and insights, would create prospects to improve their communities’ literacy about SDGs. This could be happening by drawing on comprehensive policies in different systems using trans/inter disciplinary collaboration approach and youth active participation to push on the levers of technical, political, and economic change towards the SDGs [3]. Moreover, there exists a need to help and support the generation Z to create positive changes in their social environments and food systems by providing them opportunities to become *citizen scientists* by participating in science communication and journalism activities [47]. More specifically, the digitalization of science communication and journalism operations enable greater communication of SDGs agenda among experts, and between these young citizen scientists and the lay public, which is expected to have a substantial and sustained effect on communities’ sustainable literacy over the short and long term [3, 47].

**Fig. 6:**
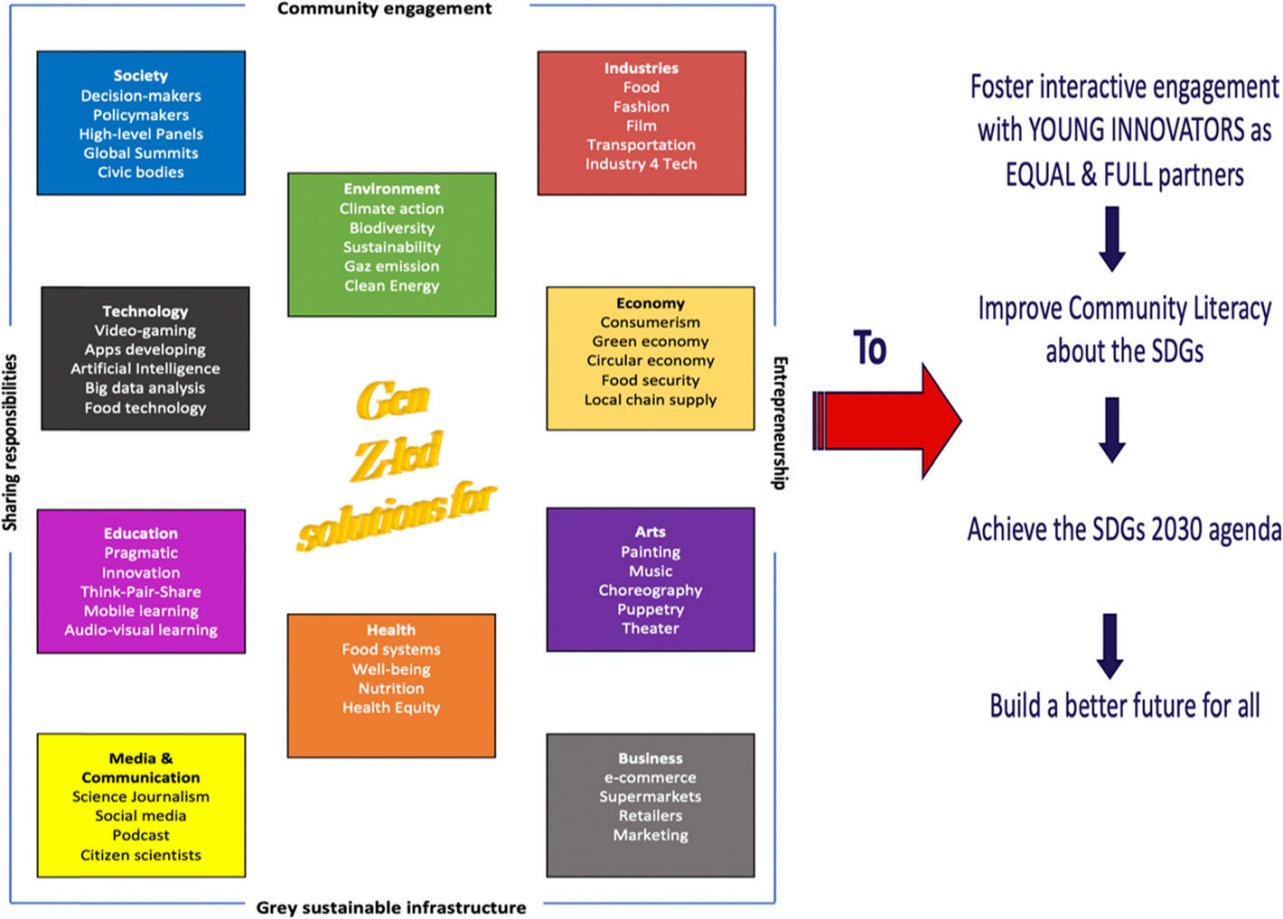
A Futuristic change-maker paradigm: Generation Z role in the SDGs 2030 agenda

Most of the works published so far refer to research, assembly reports, NGOs initiatives, and development of activities in the public, private and civic sectors [2, 6, 11, 13, 23]. To our knowledge, none of these programs used a theoretical framework, neither allied stakeholder efforts and responsibilities from different sectors, disciplines, and profiles. Therefore, a *Futuristic Change-Maker Paradigm* has been designed and proposed in this paper “Fig. 6” to demonstrate the generation Z-led solutions in their environments to improve community literacy about the 17 SDGs.

The *Futuristic Change-Maker Paradigm* is a strategic tool based on a timely approach to drive our globe to a solutions-oriented future through the generation Z innovations and solutions, as it brings together young solution-makers with multidisciplinary key stakeholders from different sectors, industry leaders, societies representatives, policy makers among others. The great ambition in the SDGs can be achieved efficiently through several types of cooperation, and global partnerships between multiple actors and across a broad range of areas.

The proposed paradigm comprised 10 interlinked and dynamic dimensions: society, environment, industries, technology, economy, education, health, business, media and communication, and arts. Grey sustainable infrastructure (buildings, roads, bridges, drains, etc.) is a fundamental key aspect of the total infrastructure of the dimensions (human, green, and blue infrastructures) that supports this paradigm. It underpins our ecosystems and circular economy in several ways and areas especially when it is strategically managed by accountable entrepreneurship, creative community engagement and transparent shared responsibilities between all communities’ members.

The inclusion of generation Z in each of these dimensions and sub-dimensions is crucial for reaching the SDGs agenda 2030. Therefore, meeting up this young generation to support their implementation efforts and compliance to create solutions in these 10 diversified dimensions is crucial to deliver many socioeconomic, health and environmental benefits. Ideally, the proposed *Futuristic Change-Maker Paradigm* will perform better than the traditional methods of SDGs implementations when we simulate the impact of generation Z on their environments, and we identify the most feasible options to involve them.

With careful attention to all generation Z attributes and considerations mentioned in the previous section, SDGs workforces at local, national, and global levels can appropriately encourage and guide this generation so that they can be a central player in the 10 interlinked dimensions of the paradigm and further contribute to their communities’ literacy about SDGs [35]. For instance, by organizing youth-centric workshops and seeking smart opportunities to integrate them in the decision-making processes related to these dimensions we can accelerate the progress on the agenda 2030.

Because young people are on the frontlines of the struggle to build a better future for all, they should be full partners in all SDGs efforts, implementations, and evaluation activities. This multifunctional integrated paradigm can be adapted and adopted by countries as the main pillar to achieve the SDGs 2030 agenda. It presents opportunities for: 1) empowering the world’s largest generation and builders of the future, 2) a constructive dialogue between all key stakeholders including youth, and 3) a resourceful multi-sectoral collaboration for the use of innovation and technology. In this sense, creative engagement of generation Z with inter-disciplinary partners such as business, science, and policy, amplifies sustainable literacy messages and enables action leading to planetary change and influence at widespread scale across all communities [26].

## Discussion

Various channels of youth participation can be associated with different levels of social and communities’ commitment. In this sense, this article; 1) confirms that youth could play an active role in utilizing state-of-the-art opportunities to address the established current UN-SDGs agenda, and 2) suggests how communication and science journalism can be conceptualized as a development intervention, where generation Z can act as *citizen scientists* and spread awareness about the SDGs in their communities*,* to go beyond the 2030 agenda. For instance, they can help to disseminate awareness about the usage of novel biosensors to detect the impact of the excessive use of insecticides in food production causing environmental pollution, toxic food contamination, and several hazards to food systems and human health [48].

This research contributes to scientific knowledge in 2 dimensions. On the one hand, it analyzes the concept of SDGs within the local, national, and international structures. On the other hand, the review incorporates a conceptual paradigm that will allow future researchers, decision- and policy- makers to develop and implement activities aimed at promoting community literacy about SDGs with the help of youth generation, in particular the generation Z.

In the future, the comprehensive paradigm proposed in this review should be evaluated in terms of practical implementation, monitoring targets, and measuring progress in different communities and contexts. Moreover, the following recommendations of future implications are drawn from the research work developed:

1. Building a ground of confidence to guarantee a *top-down support*: It is important that actions to promote sustainability literacy about the SDGs objectives in different communities have sufficient protection from the leaders and representatives of each dimension of the paradigm.
2. Creating collaborative action teams: In this way, effective collaboration between youth and all designated representatives of the 10 paradigm’s dimensions can contribute to the promotion of sustainability literacy and compliance with the SDGs. Therefore, the previous recommendation must be matched out with a *bottom-up approach*.
3. Crafting alliances and advocacies: Carrying out advocacy work with multisectoral key stakeholders (academic institutions, politicians, NGOs, etc.) is necessary so that this practical paradigm falls within the broad guidelines of the context where it is adopted.
4. Documenting and recording of data and activities: It is fundamental to document regularly and make inventories of all the programs developed, the challenges and opportunities experienced, and the reports released. Such essential step is highly recommended to avoid work duplication or unnoticed work because they have not been conveniently reported and compiled.
5. Finally, promoting the SDGs transversally throughout the paradigm’s dimensions should be reviewed, monitored, evaluated, and improved when and where necessary.

## Conclusion

This study recommends several innovative areas for the integration of generation Z insights and activities as a basis for exploring the potential of this generation to improve communities’ literacy and behaviors about the 17-SDGs. It also ends with a practical and theoretical consideration to build a *Futuristic Change-Maker Paradigm* that can be extrapolated to other countries in their advance towards sustainability and SMART environmental management.

## References

[1] Pavid K. What is the Anthropocene and why does it matter? National History Museum News. https://www.nhm.ac.uk/discover/what-is-the-anthropocene.html Accessed 31 Jan 2022.

[2] Food and Agriculture Organization of the United Nations. Food and Agriculture: Key to achieving the 2030 Agenda for sustainable development. FAO Report, 2016.

[3] Hamadeh S. Roadmap for future food systems and smart cities: Making the ecosystem connections and policies. In: Sustainable Energy-Water-Environment Nexus in deserts. Springer Nature Book. Switzerland: Springer International Publishing; 2022. pp. XXIII,843.

[4] General Assembly of United Nations. Transforming Our World: The 2030 Agenda for Sustainable Development. Resolution Adopted by the General Assembly on 25 September 2015. http://www.un.org/ga/search/view_doc.asp?symbol=A/RES/70/1&Lang=E Accessed 21 Jan. 2022.

[5] Nakatani H (2016). Global strategies for the prevention and control of infectious diseases and non-communicable diseases-meeting report. J Epidemiol.

[6] World Health Organization. Health in. From MDGs to SDGs. WHO Report. 2015;2015.

[7] United Nations. Sustainable Development Goals Officially Adopted by 193 Countries. United Nations News and Events, September 2021.http://www.un.org.cn/info/6/620.html Accessed 19 Jan. 2022.

[8] Hamadeh S. The new encyclopedia of nutrition: a reality after COVID-19. ANFS. 2020:1–10.

[9] Hamadeh S (2021). E-food commerce, nutrition economics and consumer behavior: before and after COVID-19. BJMS.

[10] Global Nutrition Report. Driving progress on the SDGs through better nutrition. GNR Report, 2019.

[11] Swissnex Network. Swiss- Middle East circular economy for youth initiative 2021. https://swisspavilion.org/wp-content/uploads/2021/01/Swiss-Middle-East-Circular-Economy-for-Youth-Initiative.pdf Accessed 4 Feb. 2022.

[12] United Nations. International Youth Day 12 August. https://www.un.org/en/observances/youth-day Accessed 3 Feb. 2022.

[13] United Nations-Office of the Secretary-General’s Envoy on Youth. Youthlead Innovation Festival https://www.un.org/youthenvoy/youthlead-innovation-festival-thematic-sessions/ Accessed 3 Feb. 2022.

[14] Berkup S (2014). Working with generation X and Y in generation Z period: management of different generations in business life. Mediterr J Soc Sci.

[15] Seemiller C, Grace M, Generation Z (2019). A century in the making.

[16] Loveland E (2017). Instant generation. J College Adm.

[17] Singh A, Dangmei J (2016). Understanding the generation Z: the future workforce. SAJMS..

[18] Wood S. Generation Z as consumers: Trends and innovations. Institute of Emerging Issues Report- NC State University, 2013.

[19] Kumar A, Shankar R, Aljohani N (2020). A big data driven framework for demand-driven forecasting with effects of marketing-mix variables. Ind Mark Manag.

[20] Tow P, Cooper I, Partridge I, Birch C, Harrington L. Principles of a systems approach to agriculture. In: Tow P, Cooper I, Partridge I, Birch C (éds.). Rainfed farming systems. Dordrecht: Springer Netherlands; 2011. pp. 03–43, DOI: 10.1007/978-1-4020-9132-2_1.

[21] United Nations Development Programme. Sustainability and equity: a better future for all. UNDP- Human Development Report. 2011.

[22] Sen A (1999). Development as freedom.

[23] Zamora-Polo F, Sanchez-Martin J (2019). Teaching for a better world. Sustainability and sustainable development goals in the construction of a change-maker university. Sustainability.

[24] United Nations Development Programme. UNDP Strategic Initiatives. https://www.undp.org/sustainable-development-goals Accessed 2 Feb. 2022.

[25] United Nations Development Programme (2015). Sustainable development goals booklet.

[26] Willet W, Rockstrom J (2019). Loken B, et al. commission food in the Anthropocene: the EAT-lancet commission on healthy diets from sustainable food systems. Lancet.

[27] Rickes P (2016). Generation in flux: how gen Z will continue to transform higher education space. PHEJ..

[28] Seemiller C, Grace M (2016). Generation Z goes to college.

[29] Nagy A, Kolcsey A (2017). Marketing or science. Acta Technol Dubnicae J.

[30] Twenge J (2017). iGen: why today's super-connected kids are growing up less rebellious, more tolerant, less happy- and completely unprepared for adulthood*and what that means for the rest of us.

[31] Howe N, Strauss W (1991). Generations: the history of America’s future, 1584 to 2069.

[32] Thomas M, Shivani M (2020). Customer profiling of alpha: the next generation market. J Bus Manag.

[33] Bialik K, Fry R. Millennial life: how young adulthood today compares with prior generations. Pew Research Center Report. 2019.

[34] Alter C. Youthquake: How the world will change when a new generation leads. Time Magazine- Double Issue, February 3, 2020.

[35] Chicca J, Shellenbarger T (2018). Connecting with generation Z: approaches in nursing education. Teach Learning Nurs.

[36] Shatto B, Erwin K (2016). Moving on from millennials: preparing for generation Z. J Contin Educ Nurs.

[37] Spears J, Zobac S, Spillane A, Thomas S (2015). Marketing learning communities to generation Z: the importance of face-to-face interaction in a digitally driven world. LCRP.

[38] Turner A (2015). Generation Z: technology and social interest. J Individ Psychol.

[39] Igel C, Urquhart V (2012). Generation Z, meet cooperative learning. Middle Sch J.

[40] Mccrindle- Facts and Stats. Gen Z and Gen Alpha infographic update. https://generationz.com.au/ Accessed 3 Feb. 2022.

[41] Gaidhani S, Arora L, Bhuvanesh S (2019). Understanding the attitude of generation Z towards workplace. IJMTE.

[42] Hansen J, Wyman D (2021). Beyond making a profit: using the UN SDGs in entrepreneurship programs to help nurture sustainable entrepreneurships. JICSB.

[43] Nespresso. Doing is everything: We are committed to create a good impact. https://www.nespresso.com/uk/en/commitments?name=nav&id=recycing-menu&creative=Our-Commitments&position=5-3 Accessed 4 Feb. 2022.

[44] TerraVirtua. Environmentally conscious Lebanese artist, Wael Hamadeh, is auctioning 3 artworks from his “Soldiers of Nature” collection 2021. https://blog.terravirtua.io/collecting/wael-hamadeh-soldiers-of-nature-auction/ Accessed 5 Feb. 2022.

[45] Save the children. Global Youth Leaders for Nutrition Report 2018. https://www.savethechildren.org.uk/blogs/2018/they-came-they-saw-they-conquered-global-youth-leaders-for-nutrition Accessed 5 Feb. 2022.

[46] The Economist Group (2021). AI comes of age: putting customers and employees at the heart of data-driven journeys.

[47] Könneker C, Lugger B (2013). Public science 2.0 – Back to the future. Science..

[48] Trinh K, Kadam U, Rampogu S (2022). Development of novel fluorescence-based and label-free noncanonical G4-quadruplex-like DNA biosensor for facile, specific, and ultrasensitive detection of fipronil. J Hazard Mater.

